# Effects of Corticosteroid Treatment on Olfactory Dysfunction in LATY136F Knock-In Mice

**DOI:** 10.7759/cureus.65791

**Published:** 2024-07-30

**Authors:** Fumi Ozaki, Takayoshi Ueno, Satoru Kondo, Misako Kaneda, Ichiro Mizushima, Kazunori Yamada, Marie Malissen, Bernard Malissen, Mitsuhiro Kawano, Tomokazu Yoshizaki

**Affiliations:** 1 Otolaryngology, Head and Neck Surgery, Kanazawa University Graduate School of Medical Sciences, Kanazawa, JPN; 2 Otolaryngology, Public Central Hospital of Matto Ishikawa, Hakusan, JPN; 3 Rheumatology, Kanazawa University Graduate School of Medical Sciences, Kanazawa, JPN; 4 Hematology and Immunology, Kanazawa Medical University, Uchinada, JPN; 5 Marseille-Luminy Immunology Center, Aix Marseille Université, Marseille, FRA

**Keywords:** growth-associated protein 43, olfactory neurons, igg4-related disease, olfactory marker protein, laty136f, olfactory dysfunction, corticosteroids

## Abstract

Objective: Immunoglobulin G4-related disease (IgG4-RD) is a systemic inflammatory condition affecting multiple organs, including the pancreas, salivary glands, lungs, kidneys, skin, and lymph nodes. Clinically, it is characterized by elevated serum IgG and IgG4 levels and tissue infiltration by IgG4-positive plasma cells, lymphocytes, fibrosis, and phlebitis obliterans. IgG4-RD is linked to increased Th2-dominant cytokines, contributing to eosinophilia, elevated serum IgG4, and fibrosis. A notable feature is its good response to corticosteroid therapy. To investigate the effects of corticosteroid treatment on olfactory dysfunction in LATY136F knock-in mice, which exhibited increased production of Th2-type IgG1 (the murine homolog of human IgG4) and developed multiorgan tissue lesions similar to those observed in IgG4-RD patients.

Methods: LATY136F knock-in mice (n=24) were divided into groups that received prednisolone or saline at different ages. Olfactory function was assessed using a behavioral test with cycloheximide. Histological and immunohistochemical analyses were performed to evaluate the olfactory epithelium thickness as well as the presence of mature and immature olfactory neurons.

Results: Corticosteroid-treated mice exhibited significantly improved olfactory function compared to the controls. Histological analysis revealed a significant increase in olfactory epithelium thickness and mature (olfactory marker protein-positive) and immature (growth-associated protein 43-positive) olfactory neurons in the treated groups compared with the control group.

Conclusion: Corticosteroid treatment effectively improved olfactory dysfunction and promoted olfactory epithelium regeneration in LATY136F knock-in mice, suggesting the potential therapeutic benefits of corticosteroid treatment for patients with IgG4-RD experiencing olfactory dysfunction. However, further research on topical nasal steroid therapy in untreated patients is warranted. The results support further investigation into topical nasal steroid therapies for treating olfactory dysfunction in untreated patients, potentially influencing clinical practice and patient management strategies for IgG4-RD globally.

## Introduction

Immunoglobulin G4-related disease (IgG4-RD) is a systemic inflammatory disease that affects many organs, such as the pancreas, salivary glands, lungs, kidneys, skin, and lymph nodes [1]. The clinical and histopathological features of this disease have been characterized. IgG4-RD exhibits high serum IgG and IgG4 levels and is characterized by IgG4-positive plasma cell infiltration, lymphocytic infiltration, fibrosis, and phlebitis obliterans in the tissue [2,3]. IgG4-RD causes an increase in Th2-dominant cytokines in lesions. These cytokines contribute to eosinophilia, elevated serum IgG4 levels, and fibrosis progression. In addition, a good response to corticosteroids is a major feature of IgG4-RD. Most patients with IgG4-RD respond to corticosteroid therapy, including partial responses.

In otolaryngology, salivary gland tumors, such as Mikulicz’s disease and Küttner’s tumors, are well established. Recently, the rhinology of IgG4-RD has focused on paranasal sinusitis [4] and olfactory dysfunction in Mikuliz’s disease [5]. We also reported olfactory dysfunction in IgG4-RD; 52% of patients with IgG4-RD exhibited olfactory dysfunction [6]. However, the etiology of this condition remains unknown. Histological evaluation by biopsy of the olfactory mucosa of a patient with IgG4-RD is desirable to elucidate the pathology; however, the small area of the human olfactory epithelium complicates analysis because of the possibility of irreversible changes. Therefore, we used LATY136F knock-in mice, an IgG4-RD model, to assess the mechanism underlying olfactory dysfunction in IgG4-RD.

The adaptor protein Linker for activation of T cell (LAT) is a key signaling hub used by the T cell antigen receptor. Mutant mice expressing loss-of-function mutations affecting LAT and including a mutation in which tyrosine 136 is replaced by a phenylalanine (LATY136F) develop lymphoproliferative disorder involving T helper type 2 effector cells capable of triggering a massive polyclonal B cell activation that leads to hypergammaglobulinemia G1 and E and to non-resolving inflammation and autoimmunity [7].

The LATY136F knock-in mouse model, characterized by a mutation in the LAT protein, exhibits features similar to IgG4-RD. These mice develop Th2-type IgG1 production, tissue lesions, inflammation, and fibrosis in multiple organs. Corticosteroid treatment effectively reduces inflammation in these mice, mirroring the response seen in IgG4-RD patients. Therefore, LATY136F knock-in mice serve as a promising model for studying human IgG4-RD [8]. We previously demonstrated that olfactory disturbances occur in LATY136F knock-in mice [9]. We also observed thinning of the olfactory epithelium, a decrease in mature olfactory neurons, a decrease in immature neurons in mice, and a suspected disorder in olfactory epithelial turnover.

This study aimed to evaluate the effect of corticosteroid treatment on the clinical and histopathological findings in LATY136F knock-in mice.

## Materials and methods

Mice

We used LATY136F knock-in mice, where the tyrosine residue at position 136 is replaced with phenylalanine (n=24; 17 males and seven females). We and others have generated knock-in mice to study the contributions of individual tyrosines of LAT to signal transduction and T cell development [7,10]. LATY136F knock-in mice develop lymphoproliferative disorders and hypergammaglobulinemia [10,11]. Mouse IgG1 is induced by Th2 cytokines and cannot bind to C1q; therefore, it is considered a homologue of human IgG4. Thus, LATY136F knock-in mice develop multiple organ lesions similar to those observed in patients with IgG4-RD, and these lesions are also sensitive to corticosteroid therapy, as in IgG4-RD. Therefore, we used LATY136F knock-in mice as a model of human IgG4-RD [8]. LATY136F knock-in mice have lesions similar to IgG4-RD in the dura and lungs [12,13].

All animal experiments were approved by the Animal Experimentation Committee and the Gene Recombination Experiment Safety Committee of Kanazawa University (Kindai6-16125). Carbon dioxide was used to euthanize the mice. Mice were maintained at the Animal Institute of Kanazawa University under conventional conditions. They were caged in an air-conditioned room at 25±2.0°C, with light from 08:45 to 20:45 and free access to water and chow.

Corticosteroid treatment

To evaluate the preventive and therapeutic effects of steroids, mice were divided into two groups: one starting steroids at four weeks of age, and the other starting steroids at seven weeks of age. Mice were administered 20 mg/kg prednisolone or normal saline three times a week for two weeks. Each group was evaluated and dissected at seven and nine weeks of age.

Behavioral testing for olfactory function

We evaluated the olfactory function of mice using a behavioral test with a cycloheximide solution [14]. Cycloheximide has a peculiar odor and unpleasant taste in mice. After the mice experienced the smell of cycloheximide, they were kept away from it. After being deprived of water for 48 hours, mice were exposed to a bottle with 0.01% cycloheximide solution and a bottle with tap water, and their behavior was observed. A “correct” answer was defined as the choice of a bottle containing tap water. Mice were observed 10 times within 10 minutes. The general accuracy rate was calculated as the number of correct answers divided by the number of trials.

Histological and immunohistochemical analysis

After behavioral testing, the mice were euthanized for histopathological analysis at seven and nine weeks of age. To maintain the natural appearance of the tissue, the mice were fixed using a perfusion-type method. After extirpating the nasal tissue, the tissue samples were fixed in 4% paraformaldehyde overnight and decalcified with a K-CX decalcification solution for two days. Sections were dehydrated with alcohol and embedded in paraffin. Serial 3 mm-thick sections were cut from each block, dewaxed, and rehydrated. The sections were stained with hematoxylin and eosin (H&E) to evaluate inflammation, fibrous changes, and the thickness of the olfactory epithelium. The olfactory epithelial thickness of the sections was measured using the ImageJ software. Two researchers measured olfactory epithelial thickness along the septum in six randomly selected fields. The mean values for the two groups of mice were compared. Specific primary antibodies against olfactory marker protein (OMP) and growth-associated protein 43 (GAP-43) were used to detect mature olfactory and immature neurons, respectively [15,16]. Antigen retrieval was performed by heating the slides for 30 minutes in citrate buffer (pH 6.0) at 90°C, cooling for 20 minutes, and washing. Endogenous peroxidase activity was quenched with methanol and 3% H2O2 for 10 minutes, followed by incubation with Protein Block Serum (Dako, Glostrup, Denmark) to decrease nonspecific binding. The sections were then incubated overnight at 4°C with primary antibodies against OMP (diluted 1:4000, goat polyclonal, Wako Pure Chemicals Industries, Osaka, Japan) and GAP43 (diluted 1:500, rabbit polyclonal, Merck Millipore, Darmstadt, Germany). For OMP staining, sections were incubated with anti-goat antibodies for 30 minutes at room temperature, followed by incubation with an avidin-biotin-peroxidase complex system (Vectastain Elite ABC kit, Vector Labs, Newark, CA, USA) for 30 minutes at room temperature. For GAP43 staining, sections were incubated with anti-rabbit antibodies (EnVisionTM+ system, Dako, Glostrup, Denmark) at room temperature for one hour. Immune complexes were detected using 3,3’-diaminobenzidine tetrahydrochloride, and sections were counterstained with hematoxylin. The specificity of the staining reaction was confirmed using non-immune serum instead of the primary antibody. The number of positive cells per 1.0 × 1.04 µm2 was counted by two researchers at three sites. Finally, the mean values for the two groups of mice were compared.

Statistical analysis

Data are shown as means ± standard errors of the mean. Statistical analysis was performed using the Mann-Whitney U test to compare the two groups. All statistical analyses were performed using SPSS Statistics version 23.0 (IBM Corp., Released 2015; IBM SPSS Statistics for Windows, Version 23.0; Armonk, NY: IBM Corp.). Statistical significance was set at p<0.05.

## Results

Behavioral testing

**Figure 1:**
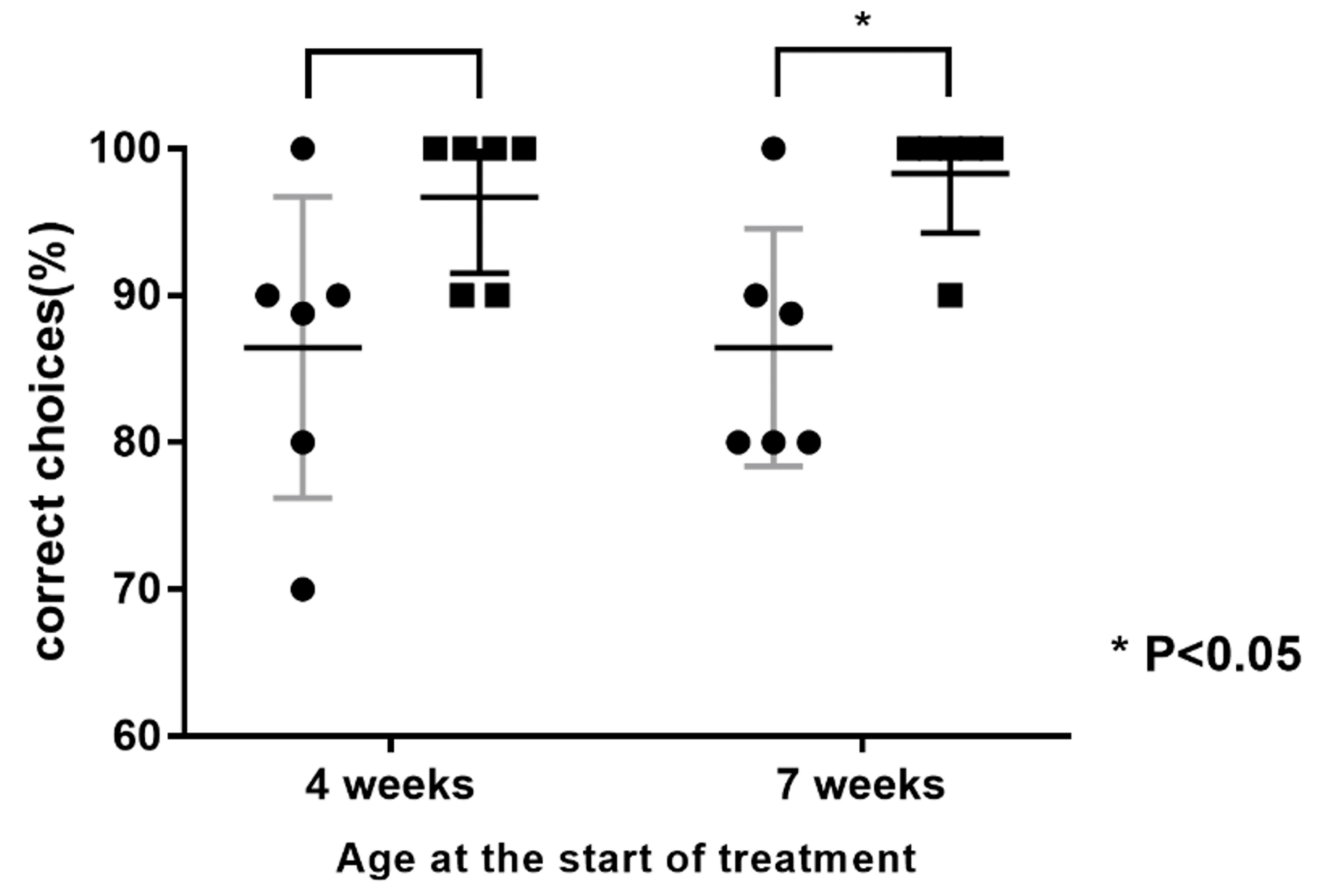
Behavioral test results evaluating olfactory function The scores of the treated group (■) were higher than those of the control group (●). The treated group received corticosteroids, while the control group received normal saline for two weeks. P-values are calculated by the Mann-Whitney U test.

The LATY136F knock-in mice made errors in terms of water selection during testing. In contrast, five out of six mice at the age of nine weeks achieved a score of 100% after corticosteroid treatment. The percentage of correct choices was 86.5±4.19% vs. 96.7±2.11% at seven weeks and 86.5±3.00% vs. 98.3±1.67% at nine weeks. The treated group at the age of nine weeks exhibited a significantly higher rate of olfactory function recovery than the control group (p<0.05) (Figure 1).

Histological and immunohistochemical findings

Olfactory Epithelium Thickness

**Figure 2:**
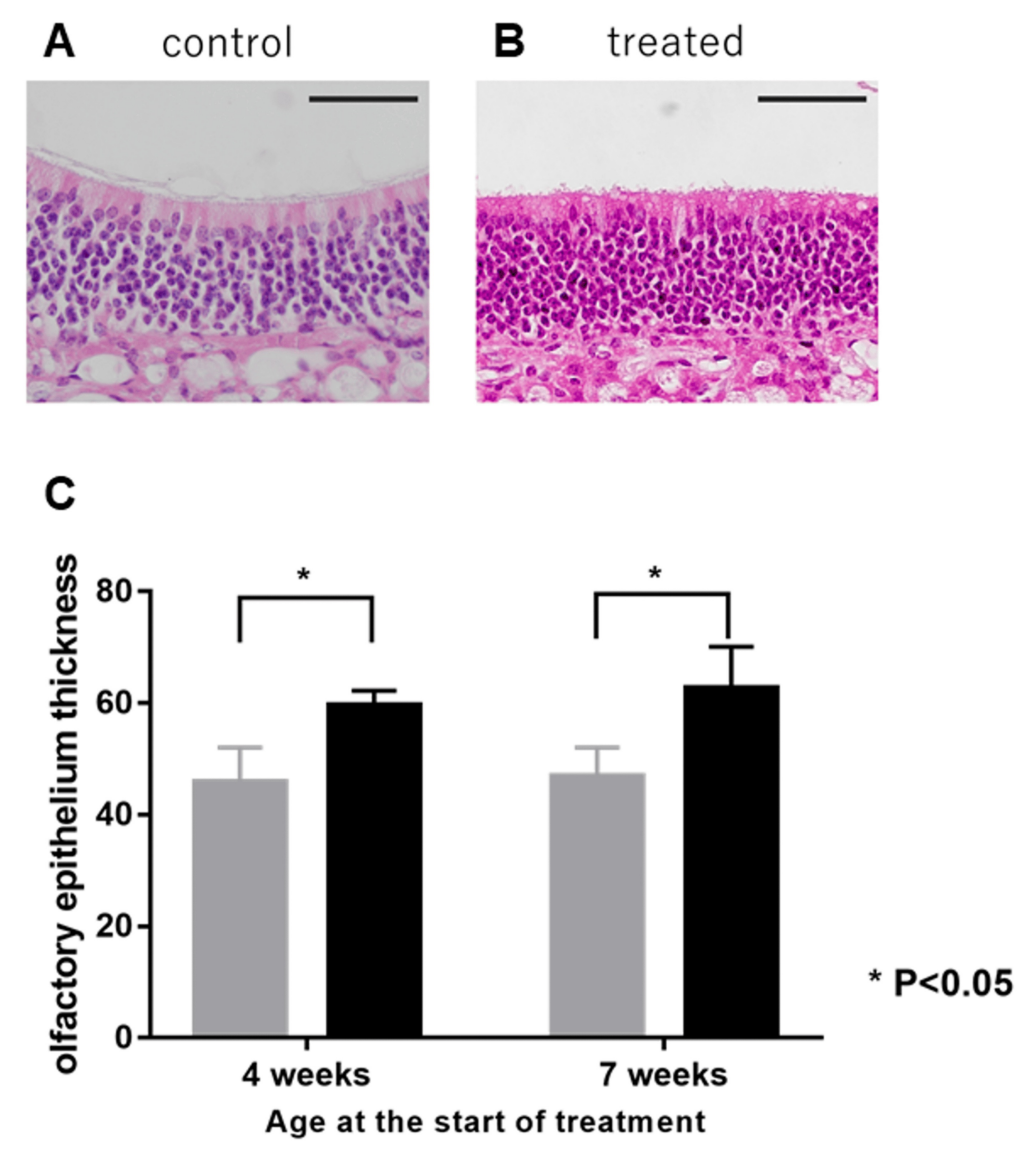
H&E staining of murine olfactory epithelium H&E staining of the olfactory epithelium of LATY136F mice was administered with normal saline (A) or corticosteroids (B) for two weeks, starting at seven weeks of age. Original magnification: ×400. Scale bar: 50 µm. Stained sections revealed that the thickness (C) of the olfactory epithelium in the treated group (black) was thicker than that in the control group (grey), regardless of their age (in weeks) (p<0.05). P-values are calculated by the Mann-Whitney U test. H&E: hematoxylin and eosin

The olfactory epithelium of LATY136F knock-in mice was thin and poor but formed a thick layer after corticosteroid treatment (Figure 2).

The mean thickness of olfactory epithelium was 46.9±2.11 µm and 62.7±3.01µm in the treated and control groups at the age of nine weeks, respectively. The olfactory epithelium was significantly thicker in the treated group than in the control group each week (p=0.0043).

OMP-Positive Cells

**Figure 3:**
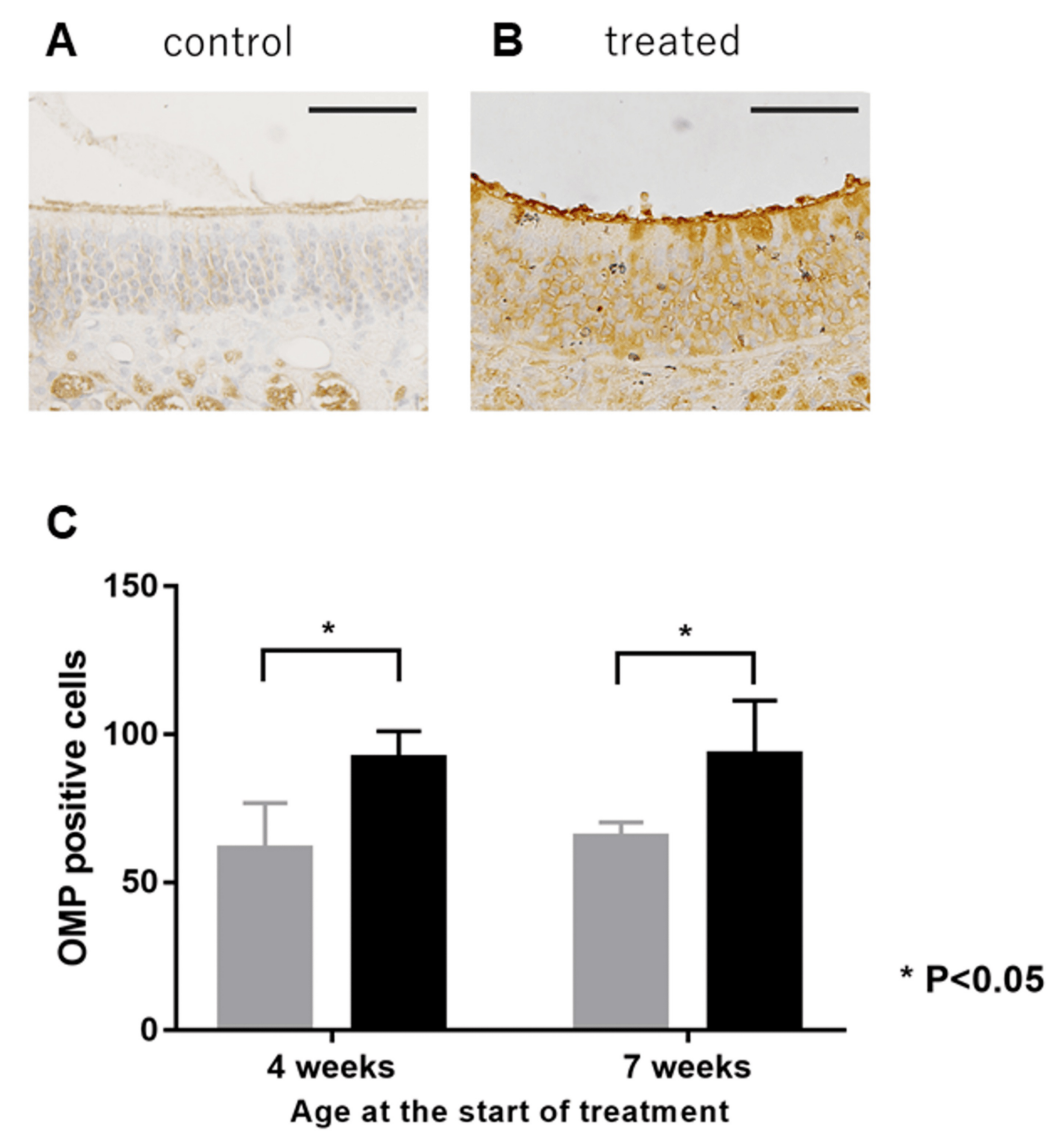
Micrographs showcasing OMP expression in the murine olfactory epithelium Micrographs showcasing OMP expression in the murine olfactory epithelium of LATY136F mice administered normal saline (A) or corticosteroids (B) for two weeks starting at seven weeks of age. Original magnification: ×400. Scale bar: 100 µm. The mean numbers of OMP-positive cells (C) on the olfactory epithelium of the treated group (black) were higher than the control group (grey). The two groups were significantly different, regardless of their age (in weeks) (p<0.05). P-values are calculated by the Mann-Whitney U test. OMP: olfactory marker protein

OMP-positive cells were present in the olfactory epithelium as mature olfactory receptor neurons and increased in number after treatment. The mean number of OMP-positive cells in the olfactory epithelium was 91.9±3.71 vs. 61.4±6.28 at seven weeks and 93.1±7.46 vs. 64.6±2.01 at nine weeks. OMP-positive cells recovered significantly after corticosteroid treatment compared to the control (p=0.0022 and p=0.0043, respectively) (Figure 3).

GAP-43-Positive Cells

**Figure 4:**
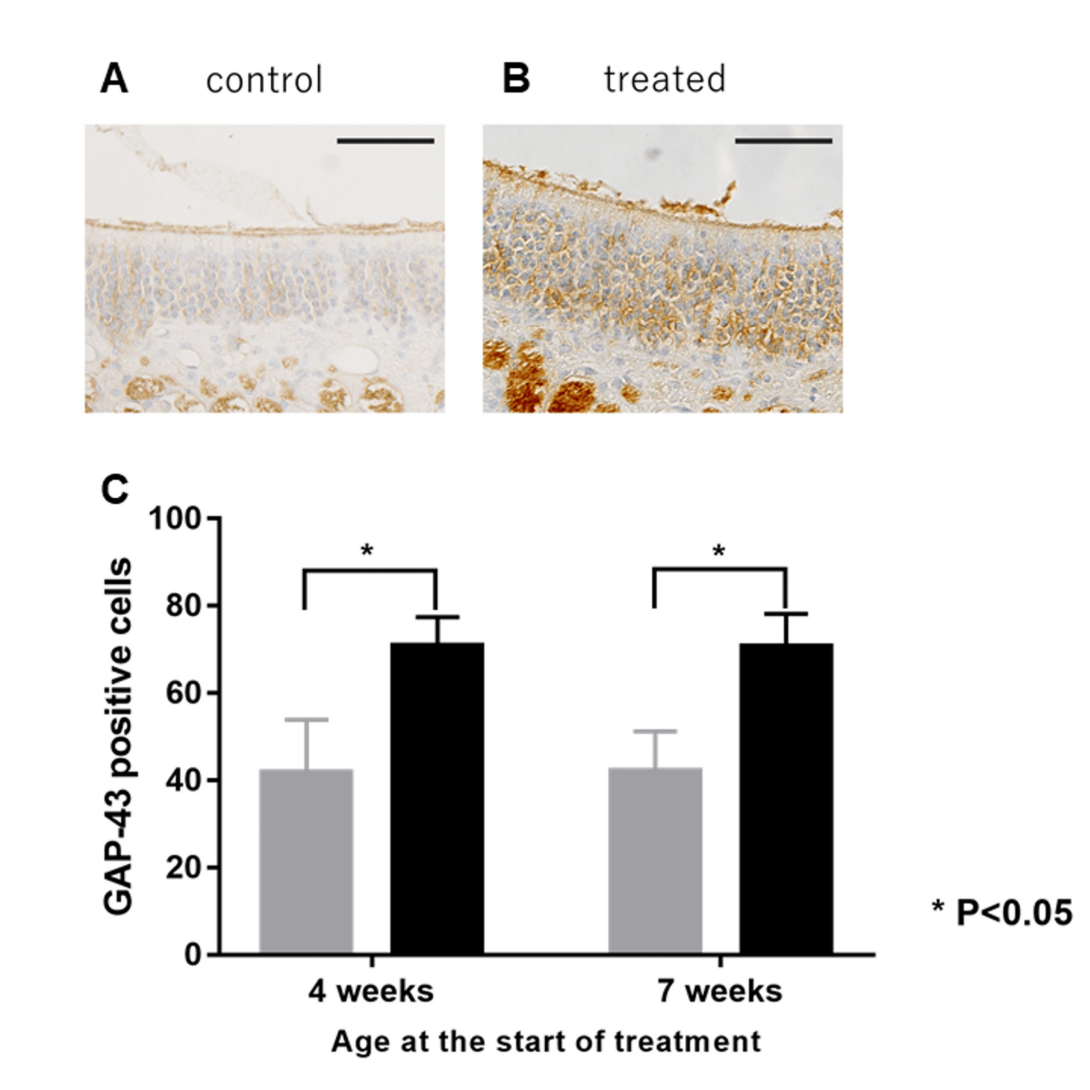
Micrographs showcasing GAP-43 expression in murine olfactory epithelium Micrographs showcasing GAP-43 expression in the murine olfactory epithelium of LATY136F mice were administered with normal saline (A) or corticosteroids (B) for two weeks, starting at seven weeks of age. Original magnification: ×400. Scale bar: 100 µm. The mean numbers of GAP-43-positive cells (C) on the olfactory epithelium of the treated group (black) were higher than the control group (grey). The two groups were significantly different, regardless of their age (in weeks) (p<0.05). P-values are calculated by the Mann-Whitney U test. GAP-43: growth-associated protein 43

GAP-43-positive cells were present in the olfactory epithelium as immature olfactory neurons and increased in number after treatment. The mean number of GAP-43-positive cells in the olfactory epithelium was 70.8±2.12 vs. 41.8±4.97 at seven weeks and 70.6±3.09 vs. 41.4±3.69 at nine weeks. These values were also significantly higher than those in the untreated group (p=0.0022 and p=0.0022, respectively) (Figure 4).

## Discussion

We evaluated the olfactory features of LATY136F knock-in mice and found that olfactory dysfunction and histopathological changes in LATY136F mice recovered following corticosteroid administration. These characteristics were also observed in patients with IgG4-RD. In a recent study, 52% of the patients with IgG4-RD also exhibited olfactory dysfunction, which recovered after corticosteroid treatment.

Corticosteroid-induced suppression of IgG4-RD-like inflammatory lesions in LATY136F mice was observed in the pancreatic, salivary gland, and kidney tissues. Additionally, we found a similar corticosteroid-induced suppression of olfactory dysfunction and the olfactory epithelium.

We previously reported that there was no significant correlation between age and olfactory dysfunction in IgG4-RD patients and LATY136F knock-in mice. LATY136F knock-in mice began to show IgG4-RD-like lesions at six weeks of age. Olfactory dysfunction in LATY136F knock-in mice was observed by at least eight weeks of age. In this study, we injected corticosteroids into LATY136F knock-in mice at four weeks of age before dysfunction was complete and observed an improvement in olfactory dysfunction. This may be due to the preventive effects of corticosteroids. In addition, the inhibition of olfactory dysfunction in seven-week-old mice, which already had olfactory dysfunction, may indicate the therapeutic effect of corticosteroids on olfactory dysfunction in LATY136F knock-in mice.

The causes of olfactory dysfunction include nasal obstruction, such as nasal polyps; olfactory neuropathy caused by olfactory epithelium damage, such as after the common cold; and central nervous system dysfunction associated with brain tumors, dementia, and other diseases. In LATY136F knock-in mice, olfactory dysfunction is histologically characterized by the thinning of the olfactory epithelium. The process of olfactory epithelial injury is not yet fully understood, but certain inflammatory cytokines may affect the olfactory epithelium since LAT136F knock-in mice exhibit a type 2/Th2 immune response.

One characteristic of IgG4-RD is that they respond to corticosteroids; however, studies also report residual histological fibrosis after corticosteroid administration [8,17]. The olfactory epithelium has the capacity for cellular replacement, which is maintained by the replenishment of mature olfactory neurons from the basal layer [18]. Therefore, if the basal cells are preserved, the olfactory epithelium can be regenerated. The olfactory epithelium of LATY136F knock-in mice was thin, but the basal cells were preserved, and there was no evidence of fibrosis. This may explain the rapid histological recovery of the olfactory epithelium in LATY136F knock-in mice following steroid administration.

Treatment of olfactory dysfunction in IgG4-RD is important because olfactory dysfunction is a symptom that impacts quality of life. Our results suggest that olfactory dysfunction in patients with IgG4-RD may improve with corticosteroid treatment. However, the treatment of IgG4-RD depends on the severity of the affected organ, and sometimes no corticosteroid treatment is chosen [19]. Whether topical nasal steroid therapy is effective in untreated patients with olfactory dysfunction remains an area for future research. Because such cases are rare, the evaluation of IgG4-RD in LATY136F knock-in mice is viable.

This study has several limitations. Firstly, the use of LATY136F knock-in mice as a model for human IgG4-RD may not fully capture the complexity of the human condition due to species differences. Secondly, the small sample size limits the statistical power and robustness of the conclusions. Thirdly, the short duration of corticosteroid treatment does not provide insights into long-term effects or potential side effects. Additionally, the study did not extensively explore the specific inflammatory pathways and cytokines involved in the changes observed in the olfactory epithelium of LATY136F knock-in mice. Finally, the lack of direct comparison with human tissue limits the generalizability of the findings.

## Conclusions

This study demonstrated that corticosteroid treatment significantly improved olfactory dysfunction in LATY136F knock-in mice, an IgG4-RD model. The treatment reversed the thinning of the olfactory epithelium and increased both mature and immature olfactory neurons. These findings suggest potential therapeutic strategies for managing olfactory dysfunction in IgG4-RD.

## References

[1] Stone JH, Zen Y, Deshpande V (2012). IgG4-related disease. N Engl J Med.

[2] Hamano H, Kawa S, Horiuchi A (2001). High serum IgG4 concentrations in patients with sclerosing pancreatitis. N Engl J Med.

[3] Umehara H (2012). A new clinical entity: IgG4-related disease (IgG4-RD) discovered in the 21st century. Intern Med.

[4] Moteki H, Yasuo M, Hamano H, Uehara T, Usami S (2011). IgG4-related chronic rhinosinusitis: a new clinical entity of nasal disease. Acta Otolaryngol.

[5] Takano K, Yamamoto M, Kondo A, Takahashi H, Himi T (2011). A clinical study of olfactory dysfunction in patients with Mikulicz's disease. Auris Nasus Larynx.

[6] Yagi-Nakanishi S, Kondo S, Kaneda M (2016). Olfactory dysfunction in IgG4-related disease. Chem Senses.

[7] Sommers CL, Park CS, Lee J (2002). A LAT mutation that inhibits T cell development yet induces lymphoproliferation. Science.

[8] Yamada K, Zuka M, Ito K (2018). LatY136F knock-in mouse model for human IgG4-related disease. PLoS One.

[9] Kaneda M, Yagi-Nakanishi S, Ozaki F (2022). Olfactory dysfunction in LATY136F knock-in mice. Auris Nasus Larynx.

[10] Aguado E, Richelme S, Nuñez-Cruz S (2002). Induction of T helper type 2 immunity by a point mutation in the LAT adaptor. Science.

[11] Genton C, Wang Y, Izui S (2006). The Th2 lymphoproliferation developing in LatY136F mutant mice triggers polyclonal B cell activation and systemic autoimmunity. J Immunol.

[12] Cui Y, Masaki K, Zhang X (2019). A novel model for treatment of hypertrophic pachymeningitis. Ann Clin Transl Neurol.

[13] Waseda Y, Yamada K, Mizuguchi K (2021). The pronounced lung lesions developing in LATY136F knock-in mice mimic human IgG4-related lung disease. PLoS One.

[14] Shiga H, Kinoshita Y, Washiyama K (2008). Odor detection ability and thallium-201 transport in the olfactory nerve of traumatic olfactory-impaired mice. Chem Senses.

[15] Baker H, Grillo M, Margolis FL (1989). Biochemical and immunocytochemical characterization of olfactory marker protein in the rodent central nervous system. J Comp Neurol.

[16] Verhaagen J, Oestreicher AB, Gispen WH, Margolis FL (1989). The expression of the growth associated protein B50/GAP43 in the olfactory system of neonatal and adult rats. J Neurosci.

[17] Mizushima I, Yamamoto M, Inoue D (2016). Factors related to renal cortical atrophy development after glucocorticoid therapy in IgG4-related kidney disease: a retrospective multicenter study. Arthritis Res Ther.

[18] Costanzo RM (1991). Regeneration of olfactory receptor cells. Ciba Found Symp.

[19] Khosroshahi A, Wallace ZS, Crowe JL (2015). International consensus guidance statement on the management and treatment of IgG4-related disease. Arthritis Rheumatol.

